# IUPACpal: efficient identification of inverted repeats in IUPAC-encoded DNA sequences

**DOI:** 10.1186/s12859-021-03983-2

**Published:** 2021-02-06

**Authors:** Hayam Alamro, Mai Alzamel, Costas S. Iliopoulos, Solon P. Pissis, Steven Watts

**Affiliations:** 1 Department of Informatics, King’s College London, 30 Aldwych, London, UK; 2 Department of Information Systems, Princess Nourah bint Abdulrahman University, Riyadh, Kingdom of Saudi Arabia; 3 Computer Science Department, King Saud University, Riyadh, Kingdom of Saudi Arabia; 4 Centrum Wiskunde & Informatica, Amsterdam, The Netherlands; 5 Vrije Universiteit Amsterdam, Amsterdam, The Netherlands

**Keywords:** Inverted repeat, Palindrome, Gaps, Mismatches, Software, IUPAC

## Abstract

**Background:**

An inverted repeat is a DNA sequence followed downstream by its reverse complement, potentially with a gap in the centre. Inverted repeats are found in both prokaryotic and eukaryotic genomes and they have been linked with countless possible functions. Many international consortia provide a comprehensive description of common genetic variation making alternative sequence representations, such as IUPAC encoding, necessary for leveraging the full potential of such broad variation datasets.

**Results:**

We present IUPACpal, an exact tool for efficient identification of inverted repeats in IUPAC-encoded DNA sequences allowing also for potential mismatches and gaps in the inverted repeats.

**Conclusion:**

Within the parameters that were tested, our experimental results show that IUPACpal compares favourably to a similar application packaged with EMBOSS. We show that IUPACpal identifies many previously unidentified inverted repeats when compared with EMBOSS, and that this is also performed with orders of magnitude improved speed.

## Background

### Context

An *inverted repeat* (IR) is a single stranded sequence of nucleotides with a subsequent downstream sequence consisting of its reverse complement [1]. Any sequence of nucleotides appearing between the initial component and its reverse complement is referred to as the *gap* (or the spacer) of the IR. The gap’s size may be of any length, including zero. In the event that the length is zero, the sequence as a whole is dubbed a *palindromic* sequence. In this event, reading from 5’ to 3’ in the forward direction on one strand reads the same as the sequence from 5’ to 3’ on the complementary strand.

IRs are a widespread occurrence [2–7] in both prokaryotic and eukaryotic genomes, and are commonly associated with a wide range of functions. Some IRs are able to extrude into DNA cruciforms, structures in which the typical double-stranded DNA denatures, and forms into intrastrand double helices or stems, consisting of complementary arms from within the same strand. At the top of each stem, unpaired loops are created from the spacer regions, and the four-way junction where the bases of the stems intersect becomes equivalent to a Holliday junction. This potential for an IR to extrude into cruciforms is dependant on the sequence composition and size of both the arms and spacer region [8]. The amount of energy required to cause such an extrusion into cruciforms via denaturing is lowered by unwinding torsional stress generated by local negative supercoiling [9, 10].

IRs are a particular class of DNA duplication in humans. Large IRs have been seen in physical maps of chromosome X, and are connected to chromosomal rearrangements and gene deletions [11–14]. The completed sequence of chromosome Y in humans, indicates the existence of many large and substantially homologous IRs, up to 1.4 Mb in size and with $99.97\%$ identity, which harbour Y-specific genes expressed in testes and considered to be essential for spermatogenesis [15]. It is apparent that gene conversion is responsible for maintaining the homology between the arms of such palindromes, and therefore the integrity of the sequence and gene functionality in the absence of meiotic recombination between homologs [16].

A common task, carried out by many international consortia, consists in providing a complete description of common genetic variation by applying whole-genome sequencing to a diverse range of subjects from multiple populations [17]. Therefore, new and qualitatively distinct computational methods and models are required to utilise the full potential of such broad datasets. One such key example of a computational paradigm shift is indicated by new representations of genomes as graphs [18] or as degenerate sequences [19] encoding the consensus of multiple sequences, marking a transition from their previous representation as regular sequences. In particular, in IUPAC encoding [20], specific symbols, referred to as *degenerate*, are employed to represent a sequence position that corresponds to a set of possible alternative nucleotides.

Various algorithmic tools and software have been published to enable the study of IRs in genomes [8, 21–23]. However, to the best of our knowledge, *the only* available tool that can meaningfully process IUPAC-encoded sequences is EMBOSS palindrome [21]. In this paper, we develop an exact and efficient tool called IUPACpal as an alternative to EMBOSS palindrome (henceforth EMBOSS). We have implemented IUPACpal to mimic the workflow, parameters and output format of EMBOSS to better enable direct comparisons in performance as well as to minimise the learning curve of using our software. We show that IUPACpal compares favourably to EMBOSS. Specifically, we show that IUPACpal identifies many previously unidentified IRs when compared with EMBOSS, and also performs this task with orders of magnitude improved speed.

### Strings

We begin with basic definitions and notation following [24]. An *alphabet* $\Sigma$ is a finite nonempty set whose elements are called *symbols*. Let $X=X[0]X[1] \dots X[n-1]$ be a *string* (or *sequence*) of length $|X|=n$ over $\Sigma$. By $\varepsilon$ we denote the *empty string*. For two positions *i* and *j* on *X*, we denote by $X[i {.\,.}j]=X[i]\dots X[j]$ the *substring* of *X* that starts at position *i* and ends at position *j*. Let *Y* be a sequence of length *m* with $0<m\le n$. We say that there exists an *occurrence* of *Y* in *X*, or more simply, that *Y* *occurs in* *X*, when *Y* is a substring of *X*. Every occurrence of *Y* can be characterised by a starting position in *X*. Thus we say that *Y* occurs at the *(starting) position* *i* in *X* when $Y=X[i {.\,.}i + m - 1]$.

The *Hamming distance* between two sequences *X* and *Y* of the same length is defined as the number of corresponding positions in *X* and *Y* with different symbols, denoted by $\delta _H(X, Y) = |\{i : X[i] \ne Y[i], i = 0, 1,\ldots , |X| - 1\}|$. If $|X| \ne |Y|$, we set $\delta _H(X, Y)=\infty$ for completeness. If two sequences *X* and *Y* are at Hamming distance *k* or less, we call this a *k-match*, written as $X \approx _k Y$.

### Degenerate strings

We use the concept of a degenerate string to model IUPAC-encoded sequences. A *degenerate symbol* ${\tilde{x}}$ over an alphabet $\Sigma$ is a nonempty subset of $\Sigma$, i.e. ${\tilde{x}} \subseteq \Sigma$ and ${\tilde{x}} \ne \emptyset$. $|{\tilde{x}}|$ denotes the size of the set and we have $1 \le |{\tilde{x}}| \le |\Sigma |$. A finite sequence ${\tilde{X}}={\tilde{x}}_0 {\tilde{x}}_1 \dots {\tilde{x}}_{n-1}$ is said to be a *degenerate string* if ${\tilde{x}}_i$ is a degenerate symbol for each $0 \le i \le n-1$. A degenerate string is built over the potential $2^{|\Sigma |} - 1$ nonempty subsets of symbols belonging to $\Sigma$. The *length* $|{\tilde{X}}|=n$ of a degenerate string ${\tilde{X}}$ is the number of degenerate symbols.

For example, ${\tilde{X}}=[\texttt {A} \texttt {C}]\, [\texttt {A}]\, [\texttt {G}]\, [\texttt {C} \texttt {G}]\, [\texttt {A}]\, [\texttt {A} \texttt {C} \texttt {G}]$ is a degenerate string of length 6 over the alphabet $\Sigma = \{\texttt {A},\texttt {C},\texttt {G}\}$ (or $\{\texttt {A},\texttt {C},\texttt {G},\texttt {T}\}$ with no occurrences of $\texttt {T}$). If $|{\tilde{x}}_i|=1$, that is $\tilde{x_i}$ represents a single symbol of $\Sigma$, we say that ${\tilde{x}}_i$ is a *solid symbol* and *i* is a *solid position*. Otherwise ${\tilde{x}}_i$ and *i* are said to be a *non-solid symbol* and *non-solid position*, respectively. For convenience we often write ${\tilde{x}}_i=\sigma$ where $\sigma \in \Sigma$, instead of ${\tilde{x}}_i=[\sigma ]$, in the case where ${\tilde{x}}_i$ is a solid symbol. Consequently, the previous example ${\tilde{X}}$ may be written as ${\tilde{X}}=[\texttt {A} \texttt {C}]\, \texttt {A}\, \texttt {G}\, [\texttt {C} \texttt {G}]\, \texttt {A}\, [\texttt {A} \texttt {C} \texttt {G}]$. A degenerate string containing only solid symbols is a *solid string* and behaves the same as a standard string of symbols, and for such strings we may omit the $\sim$ notation. In addition, a solid symbol $[\sigma ]$ and its corresponding symbol $\sigma \in \Sigma$ may be treated as interchangeable for our purposes.

For degenerate strings, the notion of symbol equality is extended to symbol equality between degenerate symbols. Two degenerate symbols ${\tilde{x}}$ and ${\tilde{y}}$ are said to *match* (denoted by ${\tilde{x}} \approx {\tilde{y}}$) if they have at least one symbol in common, i.e. ${\tilde{x}} \cap {\tilde{y}} \ne \emptyset$. Further extending this notion to degenerate strings, we say that two degenerate strings ${\tilde{X}}$ and ${\tilde{Y}}$ match (denoted by ${\tilde{X}} \approx {\tilde{Y}}$) if $|{\tilde{X}}|=|{\tilde{Y}}|$ and all corresponding symbols in ${\tilde{X}}$ and ${\tilde{Y}}$ match. Note that the relation $\approx$ is not transitive. A degenerate string ${\tilde{X}}$ is said to *occur* at position *i* in another degenerate string ${\tilde{Y}}$ if ${\tilde{X}} \approx {\tilde{Y}}[i {.\,.}i+|{\tilde{X}}|-1]$.

### Inverted repeats

For a given string *X*, we use the notation $X^R$ to refer to the *reversal* of *X*, i.e. $X^R=X[n-1]\cdots X[0]$. A *palindrome* is a string *P* which is equal to its reversal i.e. $P= P^R$.

We further use the notation ${\bar{X}}$ to refer to the *complement* of a string *X*, where the complement is defined by some bijective function $f:\Sigma \rightarrow \Sigma$. In the case of DNA alphabet, the natural choice of a complement function over the alphabet $\Sigma = \{\texttt {A},\texttt {C},\texttt {G},\texttt {T}\}$ is such that $\texttt {A} \longleftrightarrow \texttt {T}$ and $\texttt {C} \longleftrightarrow \texttt {G}$. A complement string ${\bar{X}}$ is such that ${\bar{X}}[i]=f(X[i])$ for all *i*.

Closely related to palindromes, we define an *inverted repeat* (IR) as a string that can be expressed in the form $W{\bar{W}}^R$ for some string *W*. We may generalise IRs by allowing a central gap, which we call a *gapped inverted repeat*. A gapped IR is therefore a string that can be expressed in the form $WG{\bar{W}}^R$ for some pair of strings *W* and *G* where $|G|\ge 0$. In particular note that if $G=\varepsilon$ (empty string), then the IR is ungapped.

**Fig. 1:**
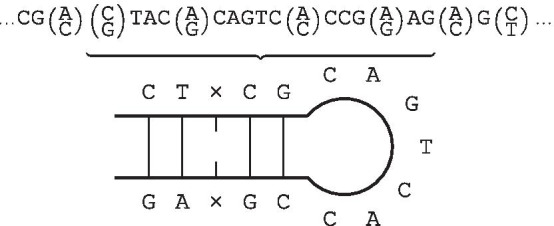
Example of an IR. The sequence CT-CGCAGTCACCG-GA is an IR with a gap of 7 and a single mismatch towards the tail ends

Finally, we may introduce mismatches by permitting the two occurrences of *W* within $WG{\bar{W}}^R$ to differ by some number of symbols, i.e. some Hamming distance. We refer to a string as a *gapped inverted repeat within k mismatches* when it can be expressed in the form $WG{\bar{W}}^R$ with $\delta _H(W, {\bar{W}}^R)\le k$. In the remainder of this paper, we use the term *inverted repeat* irrespective of whether it contains a gap, unless making the distinction is necessary. An example of an IR, which makes use of a gap and mismatches is shown in Fig. 1. This illustrates the most commonly used diagrammatic representation of IRs.

## Implementation

### IUPAC matching schemes

The International Union of Pure and Applied Chemistry (IUPAC) encoding is an extended alphabet $\Sigma ^{+}$ of symbols [20], which provides a single symbol representation for every one of the 15 possible nonempty subsets of the standard 4-symbol DNA alphabet $\Sigma = \{\texttt {A}, \texttt {C}, \texttt {G}, \texttt {T}\}$. For example, the symbol **B** represents the set {C,G,T}. This encoding provides a natural way to represent degenerate symbols using single symbols. The standard set of IUPAC symbols is $\Sigma ^{+} = \{{{{{\mathbf {\mathtt{{A}}}}}}}, {{{\mathbf {\mathtt{{C}}}}}}, {{{\mathbf {\mathtt{{G}}}}}}, {{{\mathbf {\mathtt{{T}}}}}}, {{{\mathbf {\mathtt{{R}}}}}}, {{{\mathbf {\mathtt{{Y}}}}}}, {{{\mathbf {\mathtt{{S}}}}}}, {{{\mathbf {\mathtt{{W}}}}}}, {{{\mathbf {\mathtt{{K}}}}}}, {{{\mathbf {\mathtt{{M}}}}}}, {{{\mathbf {\mathtt{{B}}}}}}, {{{\mathbf {\mathtt{{D}}}}}}, {{{\mathbf {\mathtt{{H}}}}}}, {{{\mathbf {\mathtt{{V}}}}}}, {{{\mathbf {\mathtt{{N}}}}}}\}$. The symbol **U** may also be used instead of **T**, and the symbol * instead of **N**. We therefore treat these two ambiguous pairs interchangeably.

This raises the question of how to determine complements of such IUPAC symbols, extending the current matching scheme $\texttt {A} \longleftrightarrow \texttt {T}$ and $\texttt {C} \longleftrightarrow \texttt {G}$ over $\Sigma$ to the full IUPAC alphabet $\Sigma ^{+}$. The current palindrome application within the EMBOSS package uses a method by which every IUPAC symbol is assigned a single unique complement, by first taking complements of the underlying symbols of the represented subset of $\Sigma$. For example, the complement of ${{{\mathbf {\mathtt{{B}}}}}}=\{\texttt {C},\texttt {G},\texttt {T}\}$ is ${{{\mathbf {\mathtt{{V}}}}}}=\{\texttt {G},\texttt {C},\texttt {A}\}$, and therefore ${{{\mathbf {\mathtt{{B}}}}}} \longleftrightarrow {{{\mathbf {\mathtt{{V}}}}}}$. We dub this scheme *simple complement matching*.

However, if we choose to interpret IUPAC symbols as representing a set of possibilities, then this type of matching does not take into account all possible match scenarios. Consider for example the symbol ${{{\mathbf {\mathtt{{R}}}}}}=\{\texttt {A},\texttt {G}\}$ when compared with the symbol ${{{\mathbf {\mathtt{{C}}}}}}=\{\texttt {C}\}$. Under simple complement matching, the **R** and **C** do not match, despite the fact that **R** contains G, the complement of C. Because of this potential shortcoming, we define the *degenerate complement matching* scheme over the IUPAC alphabet. Under this matching scheme, two IUPAC symbols ${{{\mathbf {\mathtt{{I}}}}}}_1$ and ${{{\mathbf {\mathtt{{I}}}}}}_2$ match if and only if there exists a pair of symbols $\sigma _1 \in {{{\mathbf {\mathtt{{I}}}}}}_1$ and $\sigma _2 \in {{{\mathbf {\mathtt{{I}}}}}}_2$ such that $\sigma _1 \longleftrightarrow \sigma _2$.

Note that the underlying algorithm of IUPACpal is independent of the matching scheme used. Though currently implemented to use degenerate complement matching, a modification of the matching matrix within the open source code permits other potential matching schemes to be defined. The matching scheme may therefore be chosen to fit the intended use case.

**Fig. 2:**
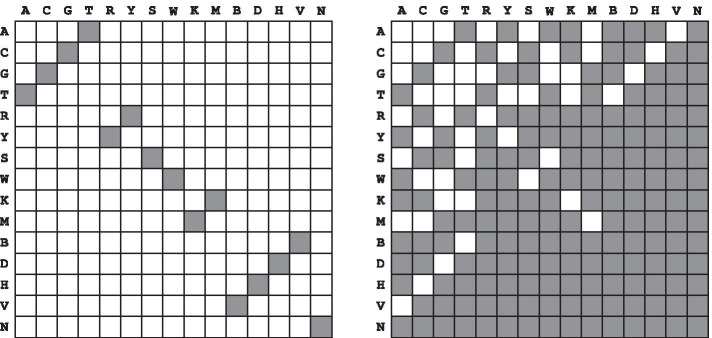
IUPAC matching schemes. Simple matching scheme on the left. Degenerate complement matching scheme on the right. White blocks indicate mismatch and filled blocks indicate match

We present a visualisation of both the simple and degenerate complement matching schemes in Fig. 2. Note that when considering sequences exclusively over the alphabet $\Sigma =\{\texttt {A},\texttt {C},\texttt {G},\texttt {T}\}$, there is no distinction between simple and degenerate complement matching.

### Algorithm

Our algorithm *exhaustively* identifies all IRs by examining each position within a sequence and determining every valid IR with its centre at that position which adheres to the given input parameters.

This process first makes use of the *kangaroo method* to create a function with the ability to identify the longest matching prefix of any two substrings of a string [25, 26]. This function of two substrings is dubbed the *longest common extension* (LCE). For a given string *X* of length *n* and two indices *i*, *j*, we define the longest common extension $\text {LCE}(X, i, j)$ as:

$$\begin{aligned} \text {LCE}(X,i,j)=\max (\{l : X[i {.\,.}i+l-1] = X[j {.\,.}j+l-1]\}\cup \{0\}) \end{aligned}$$

The kangaroo method requires an initial preprocessing of *X*, to generate indexing data structures known as the *suffix array* (SA) and the *longest common prefix* (LCP) array [27]. During preprocessing, the SA and LCP are generated twice: once for the original sequence and once for the reverse complement of the sequence. With these structures available, the kangaroo method makes it possible to find IRs with any number of mismatches with zero gap.

Our algorithm extends this capability by considering a range of possible gaps for each location in the sequence. For a given centre, the possible IRs are determined by first identifying symbols which are equidistant from the centre and are considered to mismatch.

**Fig. 3:**
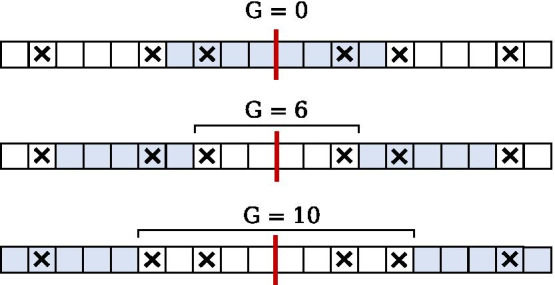
Example of 3 different IRs within a sequence. All have the same centre and are permitted 1 mismatch. The centre is marked in red. The size of the gap is given by $\texttt {G}$. Mismatching symbols are marked with $\times$. The IRs are indicated by shaded cells

Given that these mismatches can be identified, the procedure for finding IRs considers a minimal initial gap which is subsequently increased in order to reduce the number of mismatches inside the IR being considered, and thus permits a longer extension (inspect Fig. 3).

This demonstrates the principle of finding several unique IRs with the same centre by extending the gap to effectively swallow an additional mismatch, such that the IR may be extended to the position directly adjacent to the next mismatch. This extending procedure is performed repeatedly to obtain all IRs for a given centre, while taking into account the parameters specifying the maximum gap and the size range for the IR itself. The algorithm maintains efficiency by calculating only the necessary mismatch locations needed for a given set of parameters, and no more.

## Results

### Interface

We have implemented IUPACpal in $\texttt {C++}$ under GNU/Linux. IUPACpal mimics the workflow, parameters and output format of EMBOSS to better enable direct comparisons in performance. By making the key features similar and output format identical, we also minimise the learning curve of using our software. Our application requests the following parameters: input file (0), sequence name (1), output file (2), minimum length (3), maximum length (4), maximum gap (5), maximum mismatches (6). IUPACpal is run with the following terminal command:

$$\begin{aligned} {{\texttt {./IUPACpal -f<0>-s<1> -o<2> -m<3> -M<4> -g<5> -x <6>}}} \end{aligned}$$

Output is given in an identical format to that of EMBOSS, in which all the discovered IRs are identified by their index locations (1-based indexing) alongside their symbol representation. An example as applied to the IR from Fig. 1 is shown below:

$$\begin{aligned}&\;\;{\texttt {4}}\;\;\;\;{\texttt {STACR}}\;\;\;\;{\texttt {8}} \\&\;\;\;\;\;\;\;\;{\texttt {|| ||} }\\&{\texttt {20}}\;\;\;\;{\texttt {GARGC}}\;\;\;\;{\texttt {16}} \end{aligned}$$

**Fig. 4:**
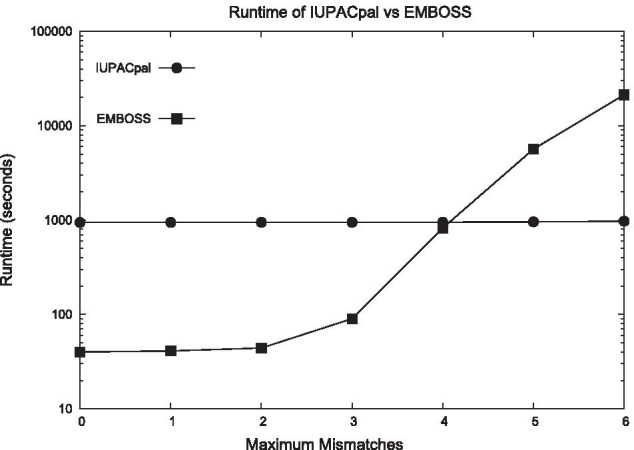
Run-time comparison. Comparison of run-time on 1,000,000 symbols of DNA. minimum length: 10. maximum length: 100. maximum gap: 100

### Run-time analysis

All experiments were conducted on a computer system using one core of Intel Core CPU i5-4690 at 3.50GHz. Both EMBOSS and IUPACpal were compiled with g++ version 6.2.0 at optimization level 3 (-O3). For a fair comparison of efficiency, we ensured that IUPACpal found at least those IRs found by EMBOSS for a given sequence. Therefore some assumptions on what constitutes a *unique* IR are replicated in IUPACpal. The IRs found by both tools are also *maximal*, i.e. cannot be extended to the left or to the right (unless further mismatches are utilised). The leftmost and rightmost symbol in any reported IR must necessarily match.

**Fig. 5:**
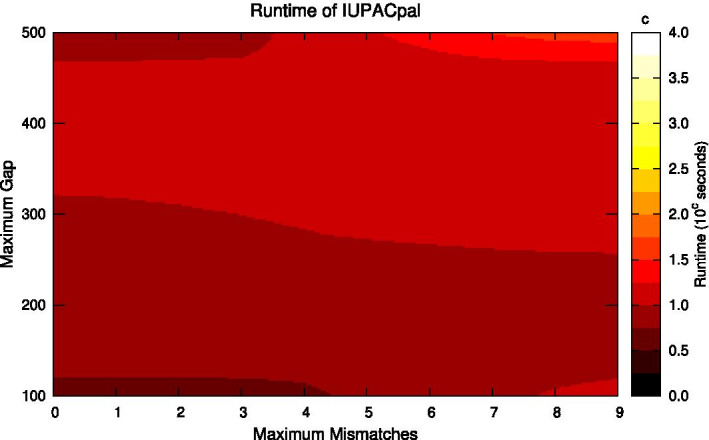
IUPACpal run-time. Run-time on 100,000 symbols of DNA for variable gap size and permitted mismatches. minimum length: 10. maximum length: 100

**Fig. 6:**
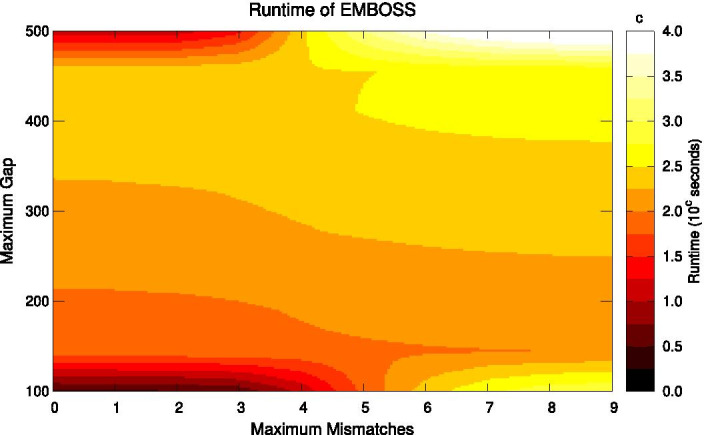
EMBOSS run-time. Run-time on 100,000 symbols of DNA for variable gap size and permitted mismatches. minimum length: 10. maximum length: 100

**Fig. 7:**
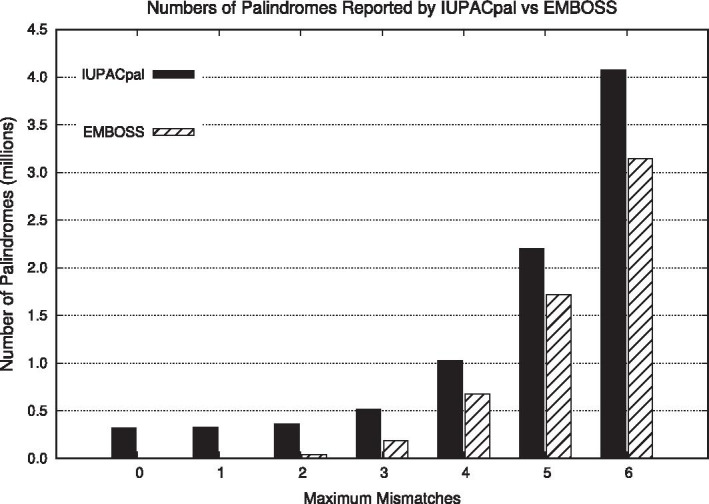
IUPACpal versus EMBOSS: Number of IRs found. Shows the number of IRs found on 1,000,000 symbols of DNA for variable number of permitted mismatches. minimum length: 10. maximum length: 100. maximum gap: 100

We ran several performance tests, providing the palindrome tool from EMBOSS and IUPACpal the same input data, and considered both their respective run-times and numbers of IRs found. We generated real IUPAC-encoded DNA sequences by combining the Genome Reference Consortium Human Build 37 (GRCh37) with the variants obtained from the 1000 Genomes Project (October 2011 Integrated Variant Set release) [17]. Specifically, we made use of chromosome X data. Results are depicted in Figs. 4, 5, 6, and 7.

In Fig. 4 we see IUPACpal performing at a consistent run-time as the maximum number of permissible mismatches increases. To the contrary, EMBOSS performs faster below 4 mismatches, yet above this threshold requires increased run-time. In practice, IUPACpal will naturally require a greater run-time for an increasing number of mismatches. However for the given parameters, the change in the order of magnitude is negligible when compared to the increase for EMBOSS in the same scenario. In fact EMBOSS required such an exponentially increasing run-time that testing was limited to no more than 6 mismatches, where EMBOSS ran in excess of 3 hours compared to IUPACpal requiring approximately 15 minutes. The run-time for IUPACpal at this number of mismatches appears to be largely dominated by the preprocessing time, rather than the increased mismatch allowance. Thus IUPACpal dominates EMBOSS in terms of speed as this overhead quickly becomes less significant.

In Fig. 5 we see IUPACpal run-time as the number of mismatches and maximum gap are both varied. This figure may be directly compared against Fig. 6, indicating a similar pattern of variation in run-time, but with significantly increased magnitude. We note some interesting details of the heat-map, such as the run-time not necessarily reducing as the permitted gap increases. For instance, within this particular testing window we see that with 0 mismatches the run-time is lowest with a gap of approximately 400 symbols. However this run-time becomes slower not only when the gap reduces to 300, but also as the gap increases to 500. However the analogous claim does not hold when keeping the gap fixed and increasing the maximum permitted mismatches. It appears that increasing mismatches always results in a slower run-time, which is to be expected when considering the algorithmic complexity of the kangaroo method.

In Fig. 6 we see EMBOSS run-time as the number of mismatches and maximum gap are both varied. We may see that the lighter colouring indicates an increase in run-time when compared to Fig. 5. Of special interest is the similar pattern of run-time distribution across the heat-map between the two figures. However we see that IUPACpal completes execution significantly faster than EMBOSS. Consider for example the run-time with 9 permitted mismatches and a maximum gap of 500, where IUPACpal requires $10^{1.5}\approx 30$s and EMBOSS requires $10^4$s to complete.

Further to the comparisons with EMBOSS, an investigation was also made of the Inverted Repeats Finder (IRF) program [8], which targets a similar problem of identifying IRs. To enable a preliminary comparison, a test run of IRF was performed in accordance with the authors’ example page [28]. Using the same testing environment as previous tests on IUPACpal, IRF was able to process human chromosome 21 (approximately 46 million base pairs) within an average of 930 s. Equivalently a rate of 50,000 DNA symbols per second. Scaling the timing tests of IUPACpal results in a speed of 130,000 DNA symbols per second. It is worth noting that the number of IRs found was relatively low within IRF (30,966 repeats found), due to the more restrictive parameters of the example run.

Let us stress that the efficacy of IUPACpal and IRF are not easily compared directly, as they utilise different paradigms of input parameters which do not naturally correspond. IRF requests a series of user defined weights, which implicitly define the IRs to be identified. In contrast, IUPACpal (and likewise EMBOSS) take a set of restraints in the minimum and maximum size of the IRs key features as input, namely the IR size and gap size. IUPACpal places emphasis on the simplicity of input parameters, and a broader matching criteria that permits a larger number of potential IRs to be identified.

### Accuracy of output

The final testing performed verified that IUPACpal is capable of exhaustively identifying at least the same IRs as EMBOSS. In addition to ensuring the usefulness of our tool, this also serves to ensure that the increases in speed performance are a result of improved algorithmic efficiency and not the result of merely solving a simpler version of the problem. A Python script was written and included as part of the software package, to verify the commonalities of the output of both tools, in addition to identifying discrepancies between the two. It was found across numerous tests that IUPACpal does indeed identify at least the same IRs as identified by EMBOSS. In a small number of cases, it was found that EMBOSS did not identify certain instances of IRs, perhaps due to considering them equivalent to some smaller IR at the same centre. However this equivalence did not seem to apply to other pairs of IRs sharing the same centre, and therefore may represent an error or small inconsistency in EMBOSS output, reported also by [23]. The results showing a comparison of the overall number of IRs found are shown in Fig. 7. Note that with a mismatch of 0, the number of IRs found by EMBOSS was relatively small (less than 1000), and thus barely registers on the figure. We see that IUPACpal consistently identifies a greater number of IRs than EMBOSS.

## Conclusions

We have presented IUPACpal, an exact and efficient tool for identifying IRs in IUPAC-encoded DNA sequences. IUPACpal has been shown to perform significantly faster than the popularly used EMBOSS tool. This speed increase appears to hold across several variations of the problem, whereby mismatches and gaps are included as additional parameters. IUPACpal also retains the ability to identify the same IRs as EMBOSS, in addition to increasing the number of IRs found. Finally, IUPACpal is designed in such a way that it could be effortlessly plugged into any pipeline, which currently relies on EMBOSS for IR identification.

## Availability and requirements

- Project name: IUPACpal
- Project home page: https://sourceforge.net/projects/iupacpal/
- Operating system(s): GNU/Linux
- Other requirements: Not applicable
- Programming language: C++
- License: GNU GPL
- Any restrictions to use by non-academics: License needed

## Data Availability

The datasets analysed and generated during the current study are available in the test_data and test_results repositories respetively: https://sourceforge.net/p/iupacpal/code/ci/master/tree/test_data/ https://sourceforge.net/p/iupacpal/code/ci/master/tree/test_results/

## Abbreviations

DNA: Deoxyribonucleic acid
EMBOSS: The European molecular biology open software suite
IR: Inverted repeat
IRF: Inverted repeats finder
IUPAC: International Union of pure and applied chemistry
IUPACpal: IUPAC palindrome tool
